# Ultranarrow Semiconductor WS_2_ Nanoribbon Field-Effect Transistors

**DOI:** 10.1021/acs.nanolett.4c01076

**Published:** 2025-01-23

**Authors:** Md. Anamul Hoque, Alexander Yu. Polyakov, Battulga Munkhbat, Konstantina Iordanidou, Abhay V. Agrawal, Andrew B. Yankovich, Sameer K. Mallik, Bing Zhao, Richa Mitra, Alexei Kalaboukhov, Eva Olsson, Sergey Kubatkin, Julia Wiktor, Samuel Lara Avila, Timur O. Shegai, Saroj P. Dash

**Affiliations:** † Department of Microtechnology and Nanoscience, Chalmers University of Technology, SE-41296 Göteborg, Sweden; ‡ Department of Physics, Chalmers University of Technology, SE-41296 Göteborg, Sweden

**Keywords:** 2D semiconductors, WS_2_, nanoribbon, transition metal dichalcogenides, TMDs, zigzag edges, diodes, field-effect transistors, crystallographically controlled nanostructuring

## Abstract

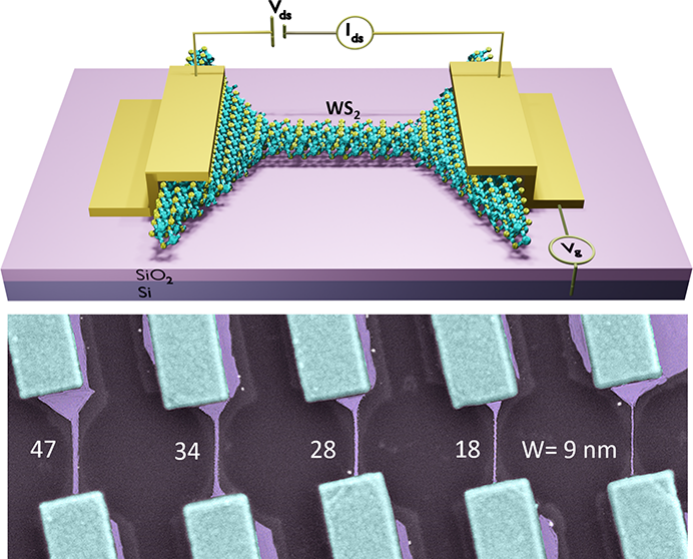

Semiconducting transition metal dichalcogenides (TMDs) have attracted significant attention for their potential to develop high-performance, energy-efficient, and nanoscale electronic devices. Despite notable advancements in scaling down the gate and channel length of TMD field-effect transistors (FETs), the fabrication of sub-30 nm narrow channels and devices with atomic-scale edge control still poses challenges. Here, we demonstrate a crystallography-controlled nanostructuring technique to fabricate ultranarrow tungsten disulfide (WS_2_) nanoribbons as small as sub-10 nm in width. The WS_2_ nanoribbon junctions having different widths display diodic current–voltage characteristics, providing a way to create and tune nanoscale device properties by controlling the size of the structures. The transport properties of the nanoribbon FETs are primarily governed by narrow channel effects, where the mobility in the narrow channels is limited by edge scattering. Our findings on nanoribbon devices hold potential for developing future-generation nanometer-scale van der Waals semiconductor-based devices and circuits.

Semiconductor field-effect transistors (FETs), celebrating their diamond jubilee, serve as the fundamental building blocks of modern computers and have transformed information technology through successful miniaturization over several decades.^1^ However, the ongoing down-scaling of conventional silicon (Si) transistors has reached its physical limits and poses significant challenges for achieving high performance and energy efficiency.^2^ In modern Fin-FET Si technology, in parallel to down scaling the channel and gate lengths, the channel widths are narrowed down to 10–20 nm, while keeping a reasonable mobility and current density.^3^ To facilitate continued progress for sub-nm technology, two-dimensional (2D) semiconducting transition metal dichalcogenides (TMDs) have attracted significant attraction due to their atomically thin body and dangling-bond-free surface.^4^ It is expected that TMD nanoribbon widths below 20 nm will be useful for future 2D FET applications in addition to chemical and biosensors because of their higher surface-to-bulk ratio and sensitivity to surface and edge chemistry.^5^ The ultimate miniaturization of channel width in TMD-based FETs will allow sustainable progress in Moore’s scaling law for high-density integration^6^ and enhanced performance beyond present Si technologies.^7−10^

The design and engineering of semiconducting TMD-based FETs and the ability to control their properties at atomic scale pose significant challenges but are of paramount importance for advances in basic science and technology.^11,12^ Recently, intensive efforts have been devoted to scaling down the channel length and width of the TMD-based FETs.^13−15^ Remarkably, devices with planar and vertical TMD FET structures featuring sub-10 nm channel length^16^ and sub-nm gate dimensions with carbon nanotube^17^ and graphene^6^ have been realized.^16−18^ However, the channel width of the state-of-the-art 2D FET devices is mainly limited to more than 25 nm^13,15,19,20^ with top-down fabrication process due to the limited resolution of lithography processes and physical etching techniques, used so far. Notably, controlling edge structures of TMD remains one of the pivotal interests because electronic properties of the edge-states have become increasingly relevant in such nanoscale devices.^21−23^

Here, we demonstrate for the first time the fabrication of sub-10 nm ultranarrow WS_2_ nanoribbons with top-down fabrication process using a crystallographically controlled wet-chemical anisotropic etching technique.^21^ The wet-etching technique allows us to go beyond the resolution of lithography with dry etching techniques and results in precise nanofabrication of ultranarrow and sharp WS_2_ nanoribbons. Interestingly, the nanoribbon junctions exhibit profound diodic current–voltage characteristics, which offer a new route to realize nanoscale diodes by the utilization of nanoribbons with different widths. Investigation of nanoribbon FETs of different widths exhibits the influence of narrow channel effects on the electronic transport properties. The mobility in the narrow channel FETs is limited by the edge and impurity scattering processes compared with the wider channels.

Figure 1a,b illustrates the device schematic and scanning electron microscopy (SEM) images of the fabricated WS_2_ nanoribbon FETs of various channel widths. A magnified high-resolution transmission electron microscopy (HRTEM) image (Figure 1c) shows the atomic structure and sharp edges of the etched WS_2_ nanoribbon. The fast Fourier transform (FFT, shown in Figure S1c) of the TEM image indicates the zigzag termination of the etched WS_2_ edge.^21^ Cardinal steps for the fabrication of nanoribbons are shown in Figure 1d, wherein WS_2_ flakes are initially exfoliated on the SiO_2_/*n*^+2^Si substrate, followed by electron beam lithography and the CHF_3_ reactive ion etching process. Finally, crystallography-controlled anisotropic wet chemical etching of WS_2_ is performed in the mixture of hydrogen peroxide (H_2_O_2_) and ammonium hydroxide (NH_4_OH) to produce WS_2_ nanoribbons down to sub-10 nm width, featuring atomically sharp zigzag edges^21^ (see the Methods in the Supporting Information).

**Figure 1:**
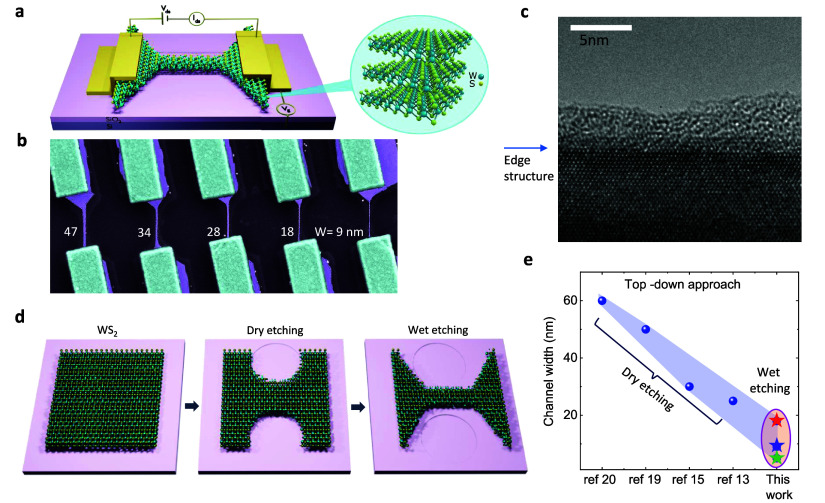
Ultranarrow nanoribbon WS_2_ devices. (a, b) Device schematic and colored scanning electron microscope (SEM) image of the fabricated multilayer WS_2_ nanoribbons of different widths (*W* = 9–47 nm) on *n*^+2^Si/SiO_2_ substrate. The measurement geometry is also shown in the schematic. (c) High-resolution transmission electron microscopy (HRTEM) image of nanoribbon reveals the crystalline edge of the WS_2_ nanoribbon after the wet etching process. The edge region has been marked by the blue arrow to observe the sharp termination of the WS_2_ crystal. The adjacent material to the WS_2_ edge is PDMS residuals from the TEM membrane transfer process. (d) Key process steps for fabricating WS_2_ nanoribbons with crystallography-controlled edges by combining dry reactive ions, wet-chemical etching, and nanopatterning processes. (e) Comparison of channel widths of our WS_2_ nanoribbon, fabricated by the wet etching method with the state-of-the-art top-down nanoribbon fabrication processes using dry etching techniques.

Figure 1e shows a comparison of our nanofabricated TMD nanoribbon channel width with the state-of-the-art results with transport measurements using top-down fabrication processes, where channel width is limited by the resolution of lithography and physical etching techniques. Electronic transport properties are investigated in WS_2_ nanoribbon FETs of different channel widths *W* = 9–47 nm in the same flake, thickness of *t* ∼ 35 nm (see Figure S2), channel length *L* ∼ 700 nm, with Ti/Au source and drain contacts and *n*^+2^Si/SiO_2_ substrate as back-gate.

The electronic properties of WS_2_ nanoribbons are characterized at room temperatures. Interestingly, the *I*_ds_ vs *V*_ds_ (*IV*) measurements show diode-like properties in nanoribbon devices where one contact is on the wider section, and the other is on the narrower section, as depicted in Figure 2a. For the 47 nm nanoribbon device, the *IV* curves at different *V*_g_ (Figure 2b) exhibit diode-like behavior, where the *I*_ds_ increases for forward bias (positive *V*_ds_), but *I*_ds_ remains negligible for reverse bias condition (negative *V*_ds_). In contrast, the wider nanoribbon (70 nm) in Figure 2c shows almost symmetric *IV* properties with nearly symmetric electrodes (see the device picture in Figure S3a). A comparison of *IV* curves of the 47 and 70 nm devices (Figure 2d) indicates that the diodic properties are more pronounced in the narrower nanoribbons with asymmetric electrodes. Interestingly, symmetric electrodes in the nanoribbon section also provide symmetric *IV* (Figure S4).

**Figure 2:**
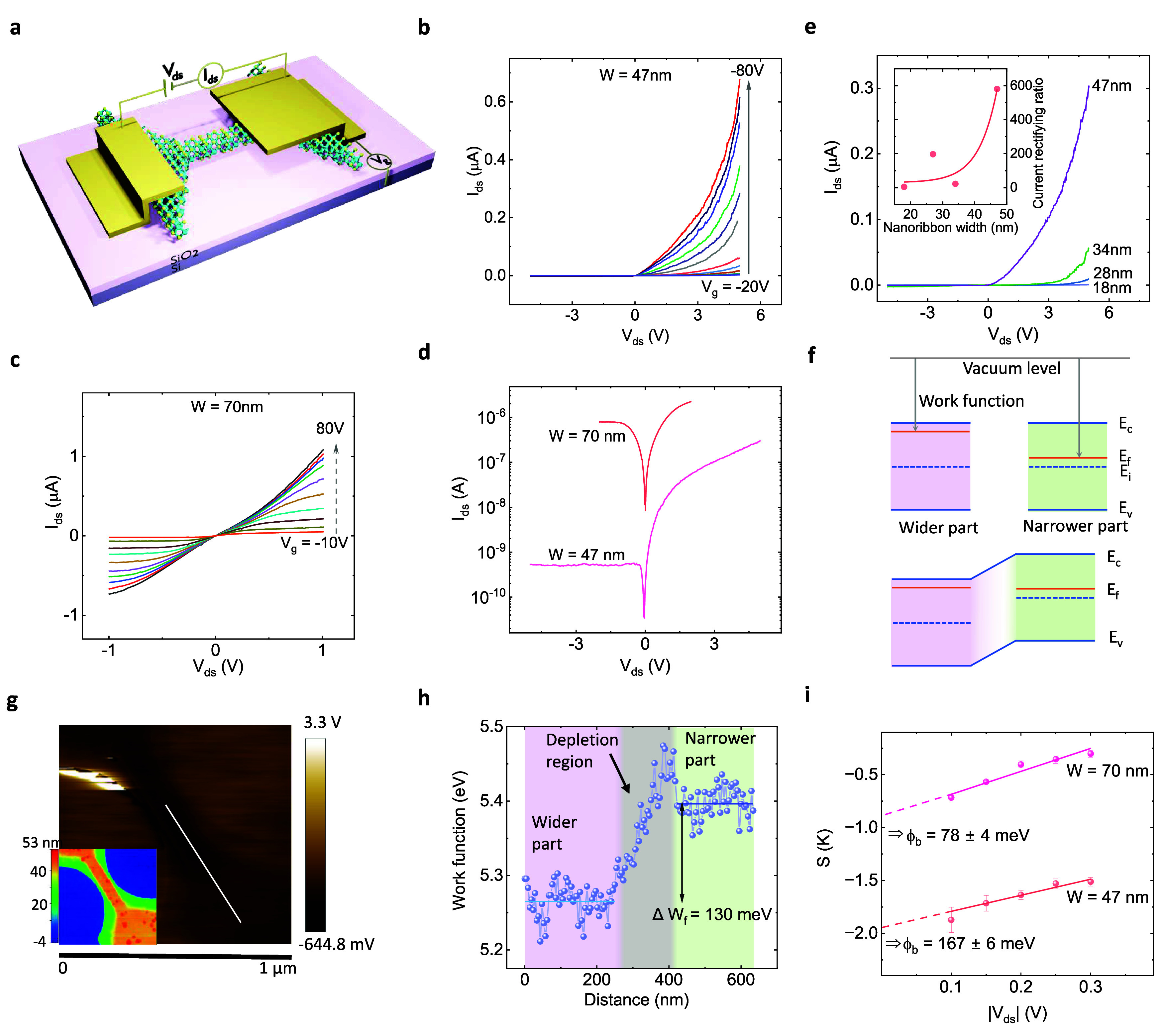
Diodic behavior of WS_2_ nanoribbon junctions. (a) Device schematic with the contacts positioned on WS_2_ nanoribbon with narrow and wider regions to measure the junction properties. (b) Diodic behavior of WS_2_ nanoribbon (*W* = 47 nm) as shown in *I*_ds_ vs *V*_ds_ plots at different *V*_g_. The same plots on the log scale are shown in Figure S5a. (c) *I*_ds_ vs *V*_ds_ plots at various *V*_g_ for *W* = 70 nm wide WS_2_ junction show less diodic behavior. Figure S5e shows the same plots on a logarithmic scale. (d) Comparison of *I*_ds_ vs *V*_ds_ properties of devices with 47 and 70 nm channel width in logarithmic scale. (e) *I*_ds_ vs *V*_ds_ plots, showing diodic behavior of devices with different nanoribbon widths (18–47 nm) at *V*_g_ = 80 V. Inset shows the current rectification ratio (*I*_ds,5V_/*I*_ds,–5V_) of the nanoribbon FET with various widths with an exponential fit (solid line) as guides to the eye. (f) Schematic band diagrams (top) of separated wider and narrower nanoribbon sections. The mismatch of Fermi level position with respect to the vacuum level is due to different doping and associated work functions in narrower and wider parts of the etched WS_2_ flake. Band diagram (bottom) of the junction with wider and narrower nanoribbon sections, where a charge depletion region emerges due to charge transfer across the junction. (g) Kelvin probe force microscopy (KPFM) profile of etched WS_2_ with wider and narrower nanoribbon sections. The respective atomic force microscopy (AFM) image of the KPFM profile section is shown for clear visualization in the inset, and the AFM analysis is provided in Figure S6. (h) The measured work function of the etched WS_2_ flake with narrower and wider sections across the white line is shown in the KPFM profile. (i) Estimated Schottky barrier height at the nanoribbons with channel widths of 47 and 70 nm.

To further confirm the diode-like properties in narrower nanoribbon devices with asymmetric electrodes, *IV* measurements were conducted for different channel widths (*W* = 18–47 nm), as shown in Figure 2e. It is also noticeable that *I*_ds_ increases with increasing W due to the decrease of channel resistivity (ρ), which is inversely proportional to W. The *IV* properties on logarithmic scale is shown in Figure S5b and the shift in *IV* curves for narrower channels can be due to higher trapped charges at the WS_2_/SiO_2_ interface.^24^ The estimated current rectification ratio and ideality factor (n) in nanoribbon FETs with various widths are shown in the inset of Figure 2e and Figure S5c, respectively.^25^ We observed the highest rectification ratio (580) in the 47 nm wide nanoribbon FET because of the contacts on the nanoribbon and wider parts that include the depletion region between the narrow and wider junction along with asymmetric contact properties. Other nanoribbon FETs show lower rectification values due to smaller depletion regions between the electrodes and less asymmetricity of the electrodes at the source and drain regions. The ideality factor (*n*) is estimated to be 4.95, 6.4, 8.95, and 22.9 for 47, 34, 27, and 18 nm nanoribbon FETs, respectively.

One hypothesis that can explain the diodic properties of WS_2_ nanoribbon is that the narrower regions have depletion of electrons due to relatively higher effects of adsorbates on the surfaces and edges^15,20^ and wet etching with H_2_O_2_ can also lead to electron depletion in WS_2_.^26^ Such phenomena can transform narrower WS_2_ nanoribbons into a lesser n-doped material in comparison to the wider section. The top panel in Figure 2f shows the schematic band diagrams of separated wider and narrower nanoribbon sections. Because of the different doping levels in the narrower and wider sections of the etched WS_2_ flake, the Fermi level position and associated work functions must also differ in narrower and wider parts in WS_2_. Furthermore, a charge depletion region must emerge due to charge transfer across the junction because of different Fermi level positions, which is presented with a band diagram in the bottom panel of Figure 2f. We can see in Figure 2d that the 47 nm channel shows higher rectification behavior because the aspect ratio of contacts touching wider and narrower sides is higher, which covers a larger depletion junction between the narrower and wider parts compared to the 70 nm wide channel.

To check the different Fermi level positions in narrower and wider parts in etched WS_2_, Kelvin probe force microscopy (KPFM) analysis is carried out, as shown in Figure 2g. Figure 2h shows the measured work function (*W*_f_) of the etched WS_2_ flake with narrower and wider sections across the white line shown in the KPFM profile in Figure 2g. The *W*_f_ calculation process from the KPFM profile is shown in the Supporting Information. We notice different *W*_f_ in the wider and narrower WS_2_ regions of about 5.27 and 5.34 eV, respectively, which is close to the reported values in multilayer semiconducting TMDs.^27,28^ The difference of *W*_f_ in narrower and wider sections is about, Δ*W*_f_ = 130 meV due to the formation of a depletion region. From both the electrical measurements and KPFM analysis, we find that the Fermi level and associated *W*_f_ are different in the narrower and wider parts of the etched WS_2_ and, thus, the depletion region across the junction results in diode-like *IV* characteristics. We have conducted KPFM analysis of nanowires of different widths (Figures S7 and S8), where we observed compatible results of *W*_f_ and Δ*W*_f_ for the nanoribbons.

Nonetheless, asymmetric electrodes in narrower and wider nanoribbon sections can also give rise to asymmetric Schottky barrier (SB) heights and contribute to such diodic behavior. The diode-like *IV* properties have been achieved by fabricating asymmetric contacts in TMDs due to the asymmetric SB because of different image force barrier lowering effects of the electrodes.^29,30^ In contrast, in the nanoribbon FETs, the diode-like *IV* properties emerge due to a potential barrier in the channel in addition to asymmetric SB at the electrodes. To check the effect of the SB at the contacts, we used the thermionic-emission model^31−34^ (Figure S3). The barrier height is found to be about, Φ_b_ = 78 ± 4 and 167 ± 6 meV for the 70 and 47 nm wide nanoribbon, respectively. The larger barrier height in the nanoribbon with a narrower channel is due to the smaller electrode-induced barrier-lowering effect in comparison to the wider channels, where the electrode-induced barrier-lowering effect is higher and results in smaller SB heights. We further estimated the SB heights at different *V*_g_ with *V*_ds_ = 5 V in the nanoribbon FETs with channel widths of 47, 34, 28, and 18 nm (Figure S9) and found consistent SB height in the nanoribbons.

To investigate field-effect transistor properties of nanoribbons, transfer characteristics (*I*_ds_ vs *V*_g_) at different *V*_ds_ are measured. The transfer characteristics of WS_2_ nanoribbon FETs with channel widths 47 and 70 nm are shown in Figure 3a,b, where the on–off ratio of the FETs is about 10^4^ (Figure S5). It is evident that with increasing *V*_g_ toward a positive value, *I*_ds_ increases due to increasing carrier concentration in the WS_2_ nanoribbon channel and *I*_ds_ increases with increasing *V*_ds_ at a fixed *V*_g_. The field-effect mobility () is estimated to be 9.5 cm^2^ V^–1^ s^–1^ for 47 nm and 24 cm^2^ V^–1^ s^–1^ for 70 nm wide channels at *V*_ds_ = 1 V, where the gate capacitance per unit area, *C*_g_ = *εε*_0_/*d*_*ox*_ = 1.15 × 10^–8^ F cm^–2^ (*d*_ox_ = 300 nm SiO_2_ structure with relative dielectric constant ε = 3.9), *L* and *W* are channel length and width, respectively, *V*_ds_ is the applied drain-source voltage, and  is the transconductance (*g*_m_). The large span of *V*_g_ is due to the thick SiO_2_ layer and can be reduced by adopting thinner oxide, high *k*-oxide, and the all-around gate electrode.^35,36^

**Figure 3:**
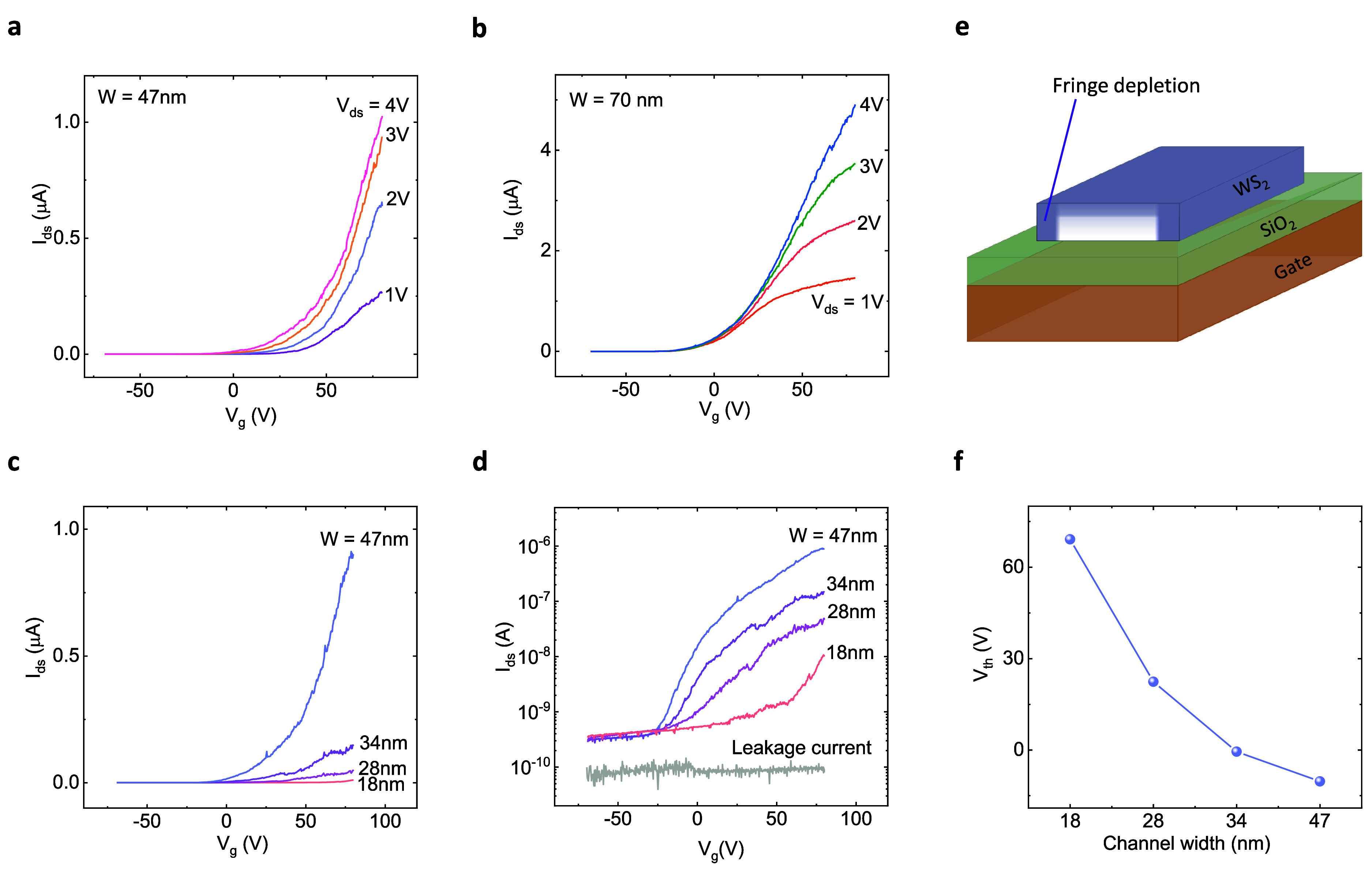
Field-effect transistor and narrow channel effect of WS_2_ nanoribbons at room temperature. (a,b) Transfer characteristics (*I*_ds_ as a function of *V*_g_) at different *V*_ds_ for WS_2_ FETs with channel widths of 47 and 70 nm, respectively. (c,d) Transfer characteristics of WS_2_ nanoribbon FETs with different channel widths *W* = 18–47 nm at *V*_ds_ = 5 V, in linear and semilog scale, respectively. The bottom gray plot in (d) denotes the gate leakage current. (e) A schematic to explain the narrow channel effect, where the fringe depletion enhances carrier depletion and consequently the change in threshold voltage (*V*_th_) in the channels. The bottom basal plane is protected by the substrate (SiO_2_); hence, a minute-scale fringing effect is expected on this nanoribbon plane. The top and edge are open to the ambient environment and are expected to have a large fringing effect on these sides. (f) The estimated *V*_th_ for WS_2_ nanoribbon FETs with different channel widths.

To observe the evolution of electrical transport properties, we compared WS_2_ nanoribbon FETs with different widths (18–47 nm) fabricated on the same flake. Figure 3c,d shows the transfer characteristics of WS_2_ nanoribbon FETs with different channel widths in the linear and semilog scale, respectively. It can be noticed that the *I*_ds_ decreases and threshold voltage (*V*_th_) shifts toward positive values with narrowing down the channel width (W). The decrease of *I*_ds_ is discussed earlier due to the increase of channel resistivity (ρ), which is inversely proportional to W. The shift of *V*_th_ can be explained by considering the narrow channel effect. In the WS_2_ nanoribbons, when the gate-induced career depletion region is comparable to the channel width, the narrow channel effect emerges in the transport measurements,^37^ as schematically presented in Figure 3e. In narrow channels, the effect of the fringe depletion region should be significant in contrast to the wider channel, as the latter has enough space for strong inversion.^37^ The origins of the fringe electron depletion in nanoribbon devices are most likely the surface absorbets^15^ and wet etching with H_2_O_2_,^26^ as edges are mainly the seeding points to adsorb external molecules and nanoparticles.^38^ The gate-induced inversion region in the narrow channel requires it to act on both fringe depletion and field depletion, which eventually increases the *V*_th_.^15,20^ The etching process provides narrow channels and, at the same time, leads to doping in the channel because the edges are susceptible to doping and defects.^38^ It would be interesting to further study the effect of different kinds of adsorbed molecules, e.g., H_2_O and O_2_, to gain more insights into the fringe effect.

Figure 3f illustrates a strong modulation of *V*_th_ caused by narrow channel effects in WS_2_ nanoribbon FETs. We estimated *V*_th_ at a fixed *I*_ds_ current of 3 nA for the corresponding transistor to simplify the data extraction and correlation process, where all the transistors are on-state.^39^ Besides, the transfer properties in our nanoribbon FETs are not similar in addition to smaller on-current and higher signal noise in the narrower channels, which complicate the *V*_th_ estimation. We observe that the *V*_th_ increases with decreasing channel width due to the narrow channel effect, since^40^
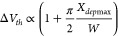
Here, *X*_*depmax*_ and *W* are the maximum depth of depletion in the WS_2_ nanoribbon and channel width, respectively. If *W* ≫ *X*_*depmax*_, then Δ*V*_*th*_ does not depend much on *W*, but for *W* ≤ *X*_*depmax*_, Δ*V*_*th*_ significantly scales with *W*, which is observed in our devices. The scaling of corresponding relative channel current (*I*_ds_/*L*) and channel mobility (μ) with the width of nanoribbons are shown in Figure S3f. These extracted parameters seem to be decreasing with reducing nanoribbon width because of more significant influences of the charge carrier depletion and edge-induced scattering mechanisms in the narrower channels.

Combining the KPFM measurements and transfer properties of the nanoribbon FETs, it can be interpreted that the nanoribbon FETs are being partially depleted by narrowing down the channel, but this charge depletion does not transform the nanoribbon into p-type from n-type or turning the nanoribbon into complete depletion condition; rather, the number of charge carriers decreases due to the fringing effect. The subthreshold swing (SS) in these nanoribbon FETs is estimated 15 and 26 V/dec for 47 and 18 nm channel width FETs, respectively. We have also measured the FETs with channel widths of 9 and 5 nm, which exhibit a further shift of *V*_th_ to higher gate voltages due to enhanced carrier depletion (see Figure. S10). The range of ON current density in our nanoribbon FETs (1.5–60 kA/cm^2^) is comparable to the recently reported nanoribbon FETs with TMDs (4–40 kA/cm^2^).^13,15,20^ However, the overall performance in our nanoribbon FETs including current density can be improved further by passivation, annealing, contact engineering, and introducing gate-all-around device design.^35,41−43^ To compare the *V*_th_ of a pristine WS_2_ FET, the transport data for a pristine WS_2_ FET transistor is shown in Figure S11. In the pristine WS_2_ FET, we estimated the threshold voltage, *V*_th_ ∼ −24.7 V, mobility, μ ∼ 44 cm^2^ V^–1^ s^–1^, and subthreshold swing SS ∼ 10 V/dec. Here, the *V*_th_ is lower than the nanoribbon channels because there is no charge depletion originating from the narrow channel effects. Furthermore, we observed p-type transport in the pristine WS_2_ FET at negative *V*_g_ due to the ambipolar transport behavior of WS_2_. Width-dependent gate voltage hysteresis between the forward and backward sweeping directions in nanoribbon WS_2_ FET due to the presence of trap states in WS_2_/SiO_2_ interface^24^ are shown in Figure S12. For comparison with literature, a recent study shows the electrical properties of TMD nanoribbons grown with a bottom-up approach,^44^ but such methods mostly provide nanoribbon clusters with a (aggregate) channel width of a few hundred nanometers,^45,46^ and in most cases, detailed electrical characterization of devices are missing.^47,48^ Furthermore, TMD nanoribbons are also produced by oxygen plasma treatment of CVD-grown TMDs, but this method is susceptible to crack formation in the layers.^49^

To understand the depletion of charge carriers and, thus, the change of *V*_th_ in the nanoribbon FETs, Density Functional Theory (DFT) calculations of the nanoribbon band structures are performed.^50,51^ DFT calculations exhibit a shift of the Fermi level position toward the valence bands, resulting in electron depletion in the reconstructed and oxidized nanoribbons compared to the pristine infinite WS_2_, which is compatible with the experimental observations (Figures S16 and S17).

We investigated the evolution of the transport properties in WS_2_ nanoribbon FETs with the temperature (*T*) for different channel widths. Figure 4(a,b,c) present the measured transfer properties in color contour plots at different temperatures for WS_2_ nanoribbon FETs of 18, 28, and 34 nm wide channels, respectively (Figure S13 presents the measured data plots). We observed an insulating behavior in the 18 nm wide WS_2_ nanoribbon channel. However, a gate- and bias-voltage induced metal-to-insulator transition in wider nanoribbons (28 and 34 nm) is observed. In the wider nanoribbon FETs, we noticed an insulating property at the low *T* range (<200 K) and metallic behavior at the higher *T* range (>200 K).

**Figure 4:**
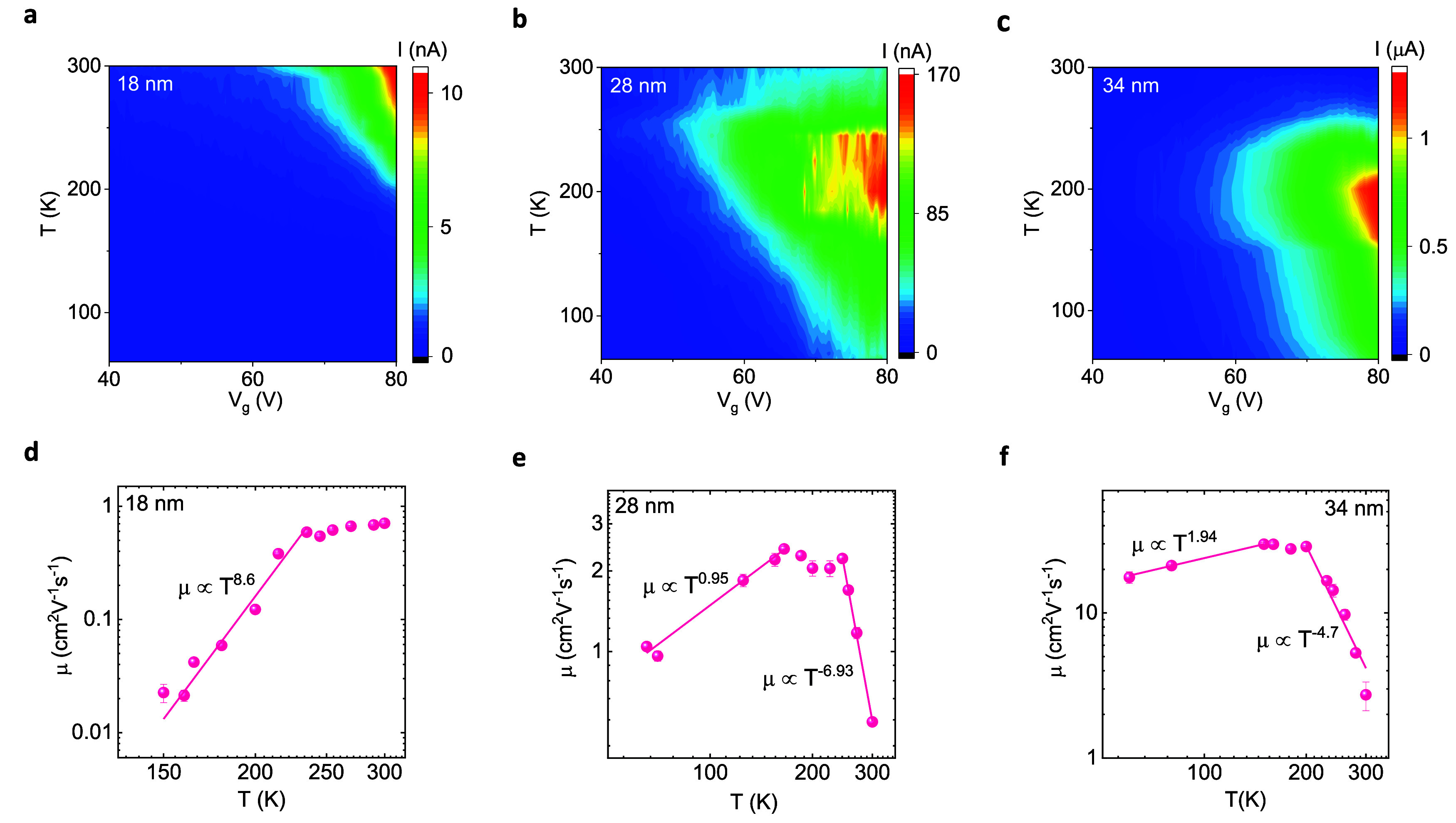
Temperature dependence of WS_2_ nanoribbon FET device parameters. (a, b, c) Color contour plots of the transfer characteristics for the WS_2_ FETs with 18, 28, and 34 nm channel widths at different temperatures with *V*_g_ in the range of 40–80 V at *V*_ds_ = 5 V. (d, e, f) Mobility μ as a function of the temperature *T* of the WS_2_ nanoribbon FETs with 18, 28, and 34 nm channel widths, along with the power-law fitting with μ ∝ *T*^*γ*^ (solid line) for different temperature ranges. The exponent γ depends on the scattering mechanisms in the nanoribbons. Error bars are estimated from the error of the fitting parameter in determining the transconductance across the channel.

The evolution of field-effect mobility (μ) with temperature is presented in Figure 4(d,e,f) for WS_2_ nanoribbon FETs with 18, 28, and 34 nm channel widths, respectively. The generic dependency of μ on *T* is fitted with μ ∝ *T*^*γ*^, where the exponent γ depends on the scattering mechanism in the nanoribbons.^52,53^ For the 18 nm nanoribbon channel, we observe an enhancement in μ with an increase in *T* at the low-temperature range and saturation in μ for higher *T* (>235 K). We estimate γ ≈ 8.6, which is mainly due to the boundary/edge or impurity scattering process in the 18 nm FET. Whereas for 28 nm FET, the γ ≈ 0.95 at the low *T* limit (<165 K) and γ ≈ −7.8 at the higher *T* limit are estimated. The limiting factor of μ at the low *T* range can be correlated to the edge or impurity scattering mechanisms, whereas phonon scattering is dominant at the higher *T* range (>250 K).^52−54^ Similarly, for the nanoribbon of 34 nm wide channel, we estimate γ ≈ 1.94 at a lower *T* range (<150 K), ascribing to the dominating edge/impurity scattering mechanism for limiting channel mobility. At the higher *T* range (>200 K), γ ≈ -4.7, consistent with the phonon scattering mechanisms limiting the channel mobility. Overall, in the wider channels, the dependencies of μ on *T* in the low-temperature regime are consistent with transport dominated by impurity scattering, whereas above ∼200 K, μ is limited by phonon scattering. The narrowest channel shows a dominant edge or impurity scattering in comparison to the wider channels, as expected, because transport properties in narrow channels are more influenced by the WS_2_ edges. Therefore, it is important to control the edge structures of TMD nanoscale devices through controlled fabrication processes,^21^ compared to fabrication via physical etching techniques. To mention, if the fringing capacitance model^46,55^ is used to calculate the mobility, the value of estimated mobility becomes smaller. However, the trend of the mobility with temperature and scattering mechanisms remains the same (see Figure S14). Temperature-dependent gate voltage hysteresis between the forward and backward sweeping directions in a representative nanoribbon WS_2_ FET is shown in Figure S15.

In summary, the realization of ultranarrow WS_2_ diodes and FETs holds great promise for semiconductor science and technology. The adapted crystallographically controlled nanostructuring process is compatible with current semiconductor manufacturing using a top-down approach. The observed tunable diode-like current–voltage characteristics in WS_2_ nanoribbons are due to the charge depletion region in the nanoribbon junction and asymmetric electrodes. The FET transport properties, such as on-current, mobility, and threshold voltage, in the extremely narrow channels are found to be governed by the narrow channel effects. The temperature-dependent mobility infers a competitive trend between phonon- and defect-mediated scattering, where the latter is dominant in narrower channels. These findings of nanoscale fabrication and unique characteristics of ultranarrow WS_2_ nanoribbon FETs open the platform for the development of nanoscale devices and investigating electronic properties of edge structures in TMDs.^22,56^

## Supporting Information Available

Detailed description of WS_2_ nanoribbon field-effect transistor (FET) fabrication method; high-resolution transmission electron microscopy (HRTEM) imaging processes; atomic force microscope image of the fabricated devices; Schottky barrier height calculations, mobility and channel currents in nanoribbon FETs; Kelvin probe force microscopy (KPFM) analysis of nanoribbons; scanning electron microscopy images of nanoribbons; *IV* and transport properties of pristine WS_2_ and nanoribbon FETs; density functional theory (DFT) calculations of WS_2_ nanoribbons (PDF)
