# Supplementary material for: Ultranarrow Semiconductor WS2 Nanoribbon Field-Effect Transistors
Source: Nano Lett. 2025 Jan 23;25(5):1750–7. doi: 10.1021/acs.nanolett.4c01076 (PMC11803707; doi:10.1021/acs.nanolett.4c01076)
Supplement: Supplementary file 1 — nl4c01076_si_001.pdf [file nl4c01076_si_001.pdf]

Supplementary Information

Ultra-narrow semiconductor WS_2 nanoribbon field-effect transistors

Md. Anamul Hoque[1], Alexander Yu. Polyakov[2], Battulga Munkhbat[2], Konstantina Iordanidou[2], Abhay V. Agrawal[2], Andrew B Yankovich[2], Sameer K. Mallik[1], Bing Zhao[1], Richa Mitra[1], Alexei Kalaboukhov[1], Eva Olsson[2], Sergey Kubatkin[1], Julia Wiktor[2], Samuel Lara Avila[1], Timur O. Shegai[2]*, Saroj P. Dash[1]*

[1]*Department of Microtechnology and Nanoscience, Chalmers University of Technology, SE-41296, Göteborg, Sweden*

[2]*Department of Physics, Chalmers University of Technology, SE-41296, Göteborg, Sweden*

Methods

For the fabrication of nanoscale diodes and FETs, first WS_2 flakes were mechanically exfoliated from bulk crystals (HqGraphene) onto SiO_2/Si substrate using the scotch-tape method, and multilayer flakes were identified by their optical contrast on the Si/SiO_2 substrate (the thickness of SiO_2 is 300 nm). We utilized the reactive ion etching and anisotropic wet etching techniques to fabricate the atomically sharp zigzag edges[1]. Anisotropic wet chemical etching of WS_2 is performed in the mixture of hydrogen peroxide (H_2O_2) and ammonium hydroxide (NH_4OH). For making nanoribbon WS_2 diodes and FETs, we prepared contacts of Ti (20 nm)/Au (90 nm) by standard electron beam lithography, electron beam evaporation, and lift-off techniques. The SiO_2/n-Si is used as a gate to control the carrier concentration in the WS_2 channel. The measured nanoribbon WS_2 FETs have a width of ~18-70 nm, a thickness of 35 nm, and a channel length of ~ 600-800 nm. The multi-channel FET is fabricated by crystallography etching WS_2 single flake via wet etching technique followed by a physical etching process. Source and drain electrodes are defined to connect multiple channels together. The electrical transport measurements were carried out in a cryostat with variable temperatures. Keithley 2612B dual-channel source meter is employed for electrical characterizations of fabricated devices. The Kelvin probe force microcopy analysis was conducted using Bruker Dimension Icon Atomic force microscope using Platinum-Iridium coated tip.

The sample for high-resolution transmission electron microscopy (HRTEM) imaging experiments was prepared by: 1. nanopatterning and etching WS_2 flakes on SiO_2/Si substrates; 2. mechanically exfoliating etched flakes onto a PDMS stamp using scotch-tape; 3. transferring the exfoliated flakes from the PDMS stamp onto a ~20 nm thick SiN TEM membrane windows (simpore.com). HRTEM experiments were performed at 60 keV on a JEOL Mono NEO ARM 200F that is equipped with a Schottky field emission gun, double Wien filter monochromator, probe aberration corrector, image aberration corrector, and Gatan Imaging Filter continuum HR spectrometer. The devices were fabricated at Myfab nanofabrication facility at Chalmers and characterized at the Chalmers Material Analysis Laboratory, CMAL.

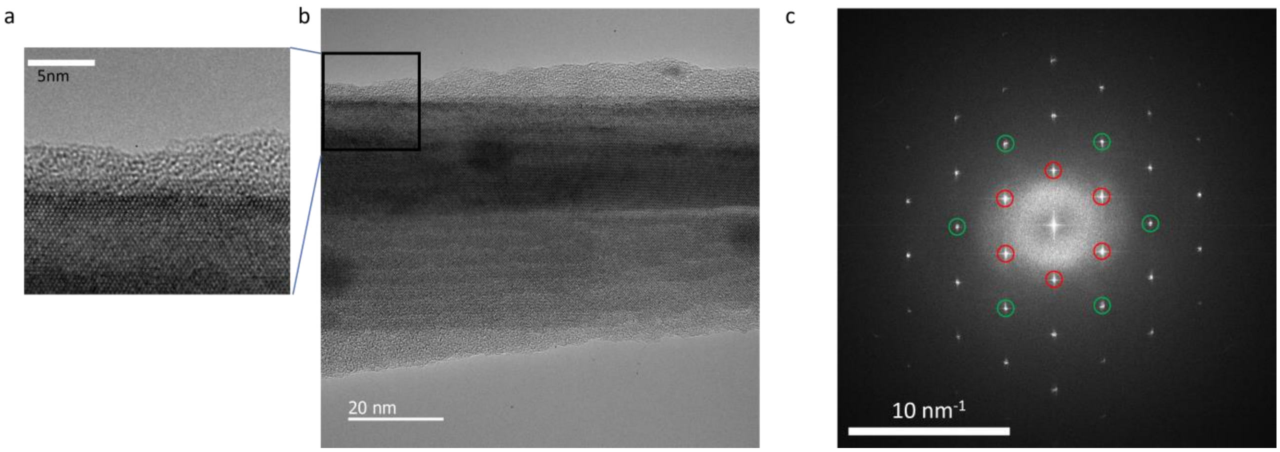

Figure S1: High resolution transmission electron microscopy (HRTEM) image of etched WS_2 nanoribbon. (a) Zoomed-in HRTEM image of a large nanoribbon, revealing pristine crystallinity and sharp edge of the WS_2 nanoribbon after wet etching process. **(b)** HRTEM image of an etched WS_2 nanoribbon. **(c)** Fast Fourier transform (FFT) of the image in (b), revealing the single crystalline nature of the WS_2 nanoribbon imaged along the [0001] zone axis. The red and green circles identify the FFT spots that correspond to the spatial frequencies of the 1100 and 1120 families of planes, respectively. The orientation between the FFT pattern and the edges of the nanoribbon in (a-b) confirms the etched surfaces are aligned with the zigzag-terminated direction[1]. HRTEM analysis with FIB cut along the nanoribbon cross-section of the various nanoribbon widths can be interesting follow-up research to study the defects and edges of the etched WS_2 nanoribbons.

Atomic force microscope image of the fabricated devices.

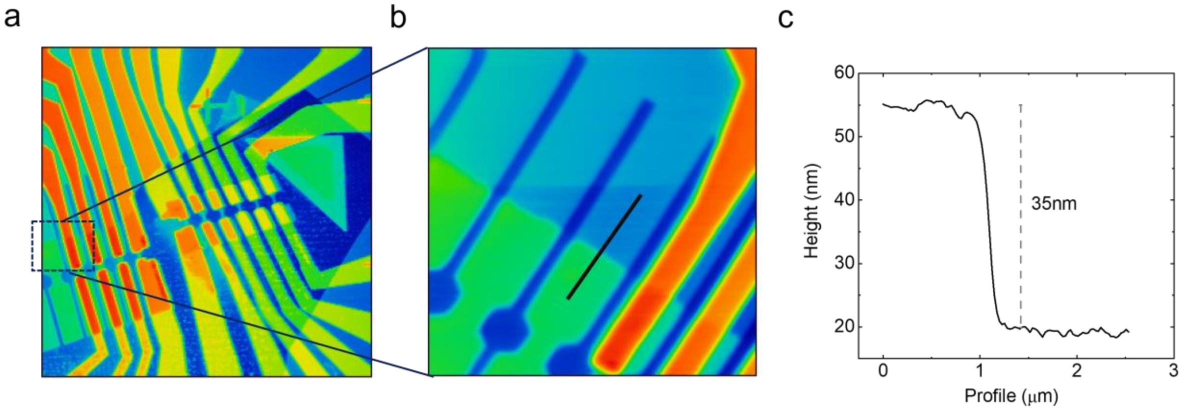

Figure S2: Atomic force microscope image. (a) Atomic force microscope (AFM) image of several nanoribbon field-effect transistors fabricated on a single flake, measured after electrical measurements were finished. **(b)** Zoom-in AFM image of a section that is used for thickness measurement of the flake. **(c)** The height profile (along the black line in (b)) shows the thickness of the WS_2 flake is about 35 nm.

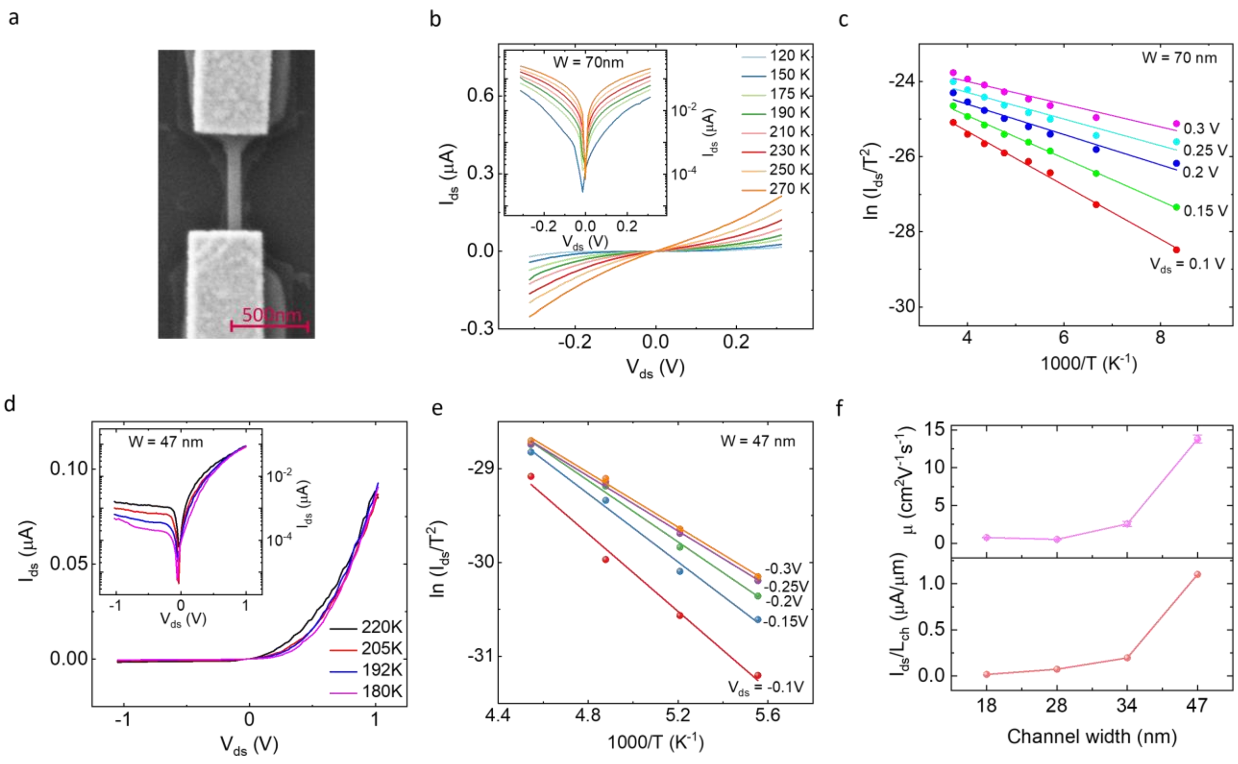

Figure S3: Schottky barrier height, mobility and channel current in nanoribbon FETs. (a) Scanning electron microscope image of the nanoribbon FET with channel width 70 nm. **(b)** I_{ds} vs. V_{ds} plots of the 70 nm wide WS_2 nanoribbon FET at different temperatures at V_g=80 V. Inset shows the I_{ds} vs. V_{ds} in log scale. **(c)** Arrhenius plots of ln(I_{ds}/T^2) with 1000/T at different V_{ds} in the nanoribbon FET with channel width 70 nm. **(d)** I_{ds} vs. V_{ds} plots of the 47 nm wide WS_2 nanoribbon at different temperatures at V_g=80 V. Inset shows the I_{ds} vs. V_{ds} on a long scale. **(e)** Arrhenius plots of ln(I_{ds}/T^2) with 1000/T at different V_{ds} in the nanoribbon FET with channel width 47 nm. **(f)** The field-effect mobility μ (top panel) and on-state current level I_{ds} (bottom panel) at V_g=80 V for the WS_2 nanoribbon FETs with different channel widths with V_{ds}=5 V.

Symmetric IV properties in nanoribbon FET

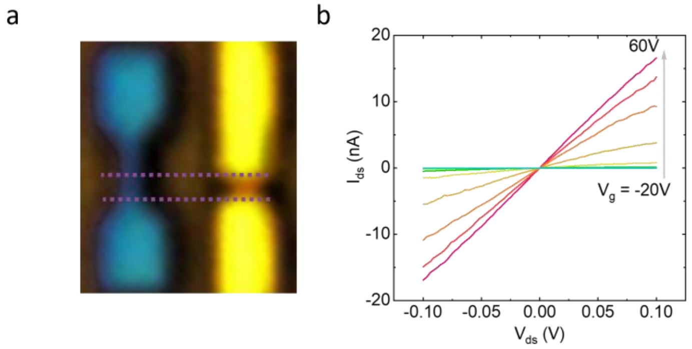

Figure S4: IV properties of WS_2 nanoribbon FET. **(a)** Microscopy image of a nanoribbon FET with symmetric electrodes. The channel width is about 150 nm of the FET, and the length is 200 nm. An adjacent etched flake is

demonstrated to depict the symmetric placement of the source and drain electrodes on the channel. **(b)** Measured IV properties of the WS_2 FET at different V_g. The IV shows symmetric behavior with bias voltage.

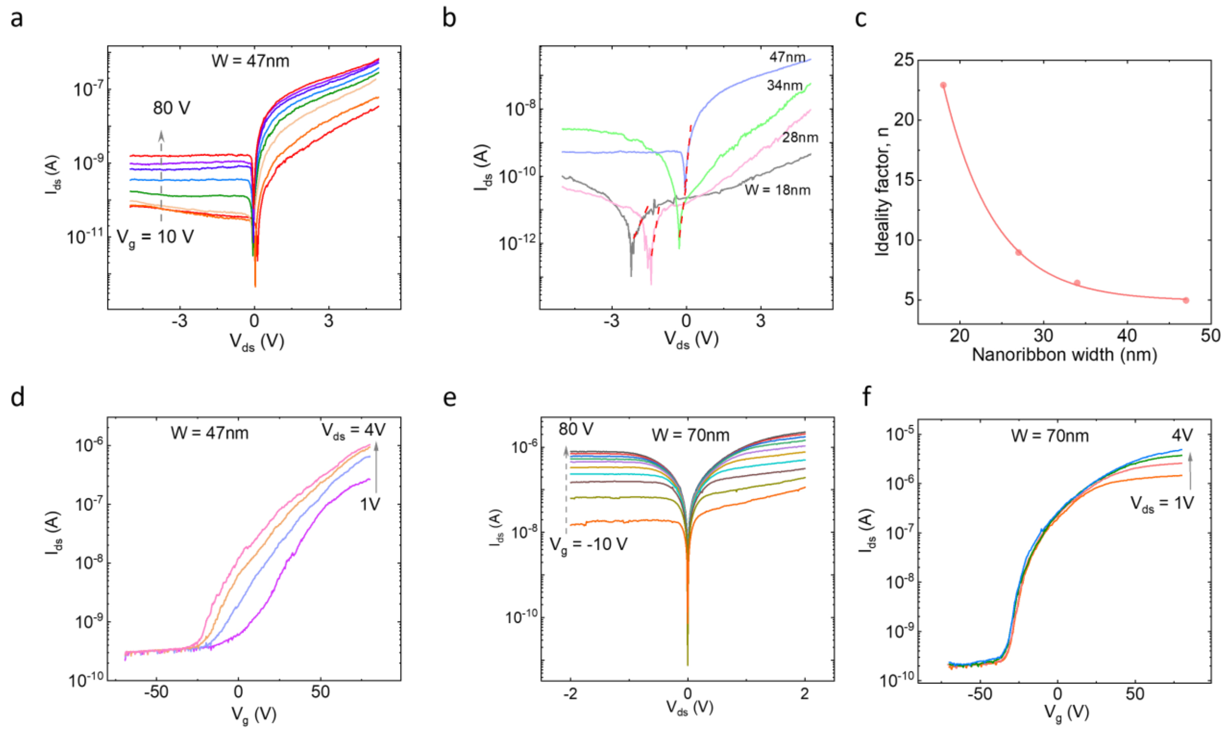

Figure S5: IV properties in nanoribbon FETs on logarithmic scale. (a) The gate-dependent diodic IV properties in 47 nm wide nanoribbon FET in logarithmic scale for clear depiction. **(b)** I_{ds} *vs.* V_{ds} plots at V_g=80 V in logarithmic scale, showing diodic behavior of WS_2 FETs with different nanoribbon widths (18-47 nm). **(c)** Extracted ideality factor (n) in WS_2 nanoribbon FETs with different widths along with an exponential fitting as guides to the eye. The n is calculated along the red lines in the IV measurements, shown in Figure S5b. **(d)** The transfer properties in the logarithmic scale of the nanoribbon FETs with a width of 47 nm. **(e)** The gate-dependent IV properties in 70 nm wide nanoribbon FET in logarithmic scale. **(f)** I_{ds} *vs.* V_g at different V_{ds} in the logarithmic scale of the 70nm wide nanoribbon FET in logarithmic scale. We would like to mention that in this manuscript, we have reported electrical measurements of one nanoribbon FET for each width (18 nm - 47 nm), that was fabricated on one flake. We measured the FETs for a long time at various temperatures, indicating the reproducibility and robustness of the device measurements and fabrication processes.

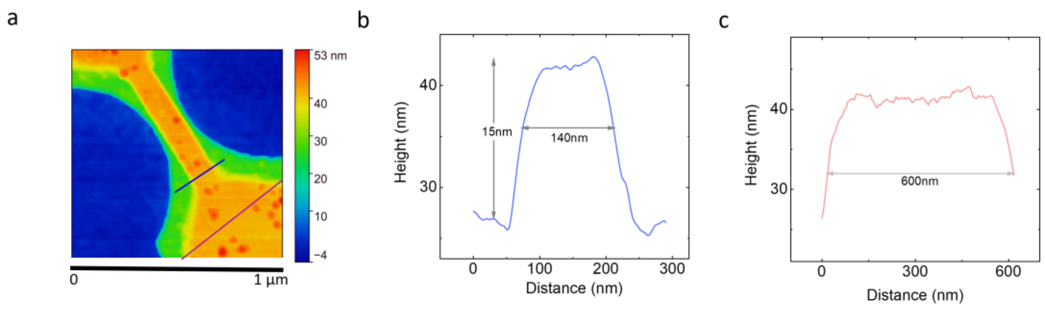

Figure S6: Atomic force microscope image of nanoribbon FET. **(a)** Atomic force microscope (AFM) image of a nanoribbon field-effect transistors. **(b)** The height profile (along the black line in (a)) shows the thickness of the WS_2 flake is about 15 nm and the width of the nanoribbon section is 140 nm. **(c)** The height profile in the wider section across the purple line and the width of the wider region is more than 600 nm.

Kelvin probe force microscopy (KPFM) analysis of WS_2 nanoribbons

We have conducted a KPFM analysis of a few nanowires of different widths (see Figure S7 and Figure S8 below). The estimated work function (W_f) and the change of work function (ΔW_f) for the nanoribbons are presented in Figure S8e and Figure S8f. In a nutshell, we have observed the highest W_f (5.55 eV) in the narrowest (50 nm wide) nanoribbon, followed by the moderately wider nanoribbon (5.37 eV) and widest ribbon shows the lowest W_f (5.17 eV). Furthermore, we observed a significant difference in work function in the narrowest nanoribbon junction (ΔW_f = 140 meV), followed by the one with moderately wider nanoribbon (140 nm) junction (ΔW_f = 130 meV) and no change in the W_f in the widest (200 nm) nanoribbon junction. To be noted, the lower W_f indicates higher n-type doping in the channel, as expected for the wider ribbon and higher W_f indicates depletion of carriers in the channel (as shown in Figure 2f in the main manuscript). This analysis confirms our findings of the electrical measurements in this article, which show that the narrower channel shows depletion of carriers in comparison to the wider regions, and a depletion region emerges between the narrow and wider nanoribbon regions. Further studies of the KPFM analysis (Figure S7b in the Supplementary Information) show the emergence of a depletion region on both sides of a nanoribbon between narrower and wider regions, which excludes any artifact-mediated KPFM analysis. Otherwise, the estimated work function (W_f) should show an increasing, decreasing, or unchanged trend across the nanoribbon if there were resolution-limiting artifacts. The estimated W_f in the nanoribbon cross-section is presented in Figure S7d of the Supplementary Information, which is consistent with the longitudinal analysis. Furthermore, the emergence of the depletion region between wider and narrower nanoribbon sections is also supported by electrical measurements.

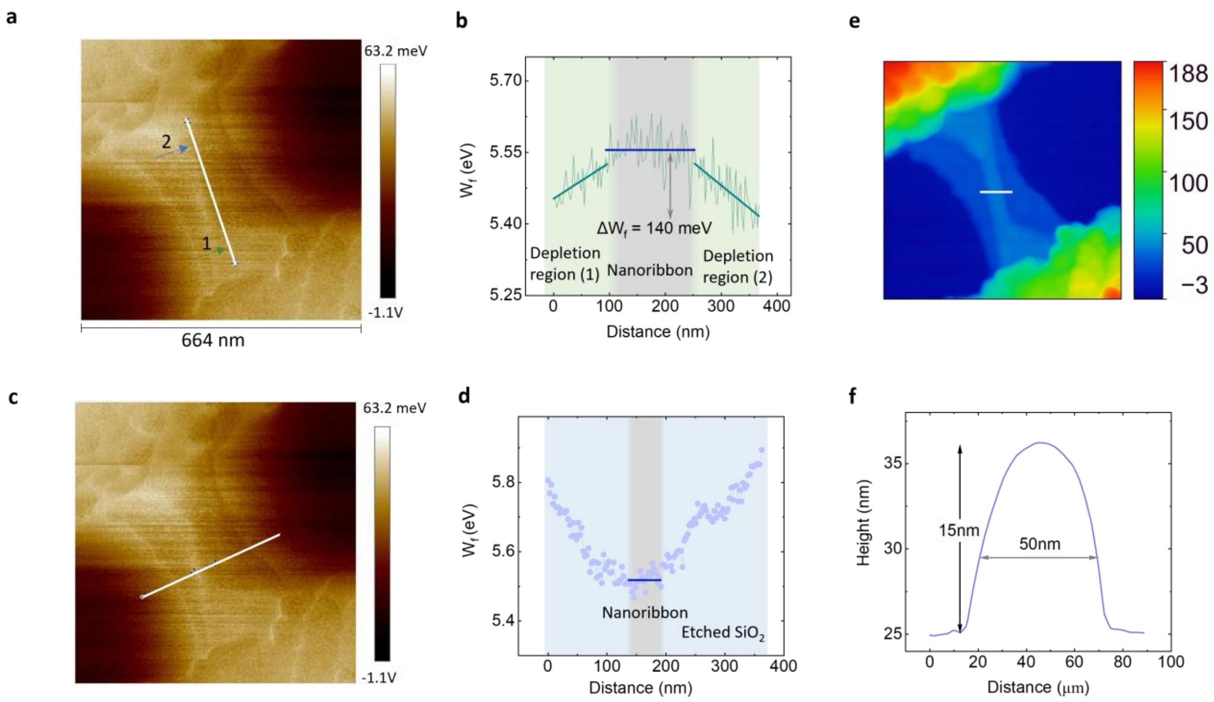

Figure S7: Kelvin probe force microscopy (KPFM) profile of nanoribbon WS_2 channel. (a) Kelvin probe force microscopy (KPFM) image of an etched WS_2 nanoribbon with wider and narrower nanoribbon sections. Regions '1' and '2' denote the junctions between wider and narrower nanoribbon sections. **(b)** The estimated work function of the etched WS_2 nanoribbon with narrower and wider sections across the white line in Figure S7a. For this nanoribbon channel, the work function (W_f) for the narrow channel is about 5.55 eV, which is a bit higher than the estimated W_f of the nanoribbon presented in Figure 2h in the main manuscript because this nanoribbon is narrower than the one presented in Figure 2h. Here, two depletion regions correspond to the regions '1' and '2' in Figure S7a. Interestingly, the W_f in the wider regions does not show any saturation values because the width in the wider regions varies from 50 nm to 150 nm, as shown in the later AMF image. The non-saturation properties of W_f in this nanoribbon differ from the analysis presented in Figure 2h in the main manuscript, where the wider section is quite larger (600 nm) compared to the nanoribbon section. **(c)** KPFM image of the etched WS_2 nanoribbon that is used to analyze W_f across the ribbon cross-section, as presented by the white line in this image. **(d)** The estimated W_f across the etched WS_2 nanoribbon cross section, as denoted by the white line. The W_f is about 5.55 eV in the nanoribbon section similar to the longitudinal analysis (Fig. S7b), but the SiO_2 shows varying W_f because of variation of etching profile that took place during physical and chemical etching under the flake. **(e)** AFM image of the nanoribbon that is used for KPFM analysis. **(f)** The height profile (along the white line in (e)) shows the thickness of the WS_2 flake is about 15 nm and the nanoribbon is about 50 nm wide and wider regions vary from 50 nm – 150 nm.

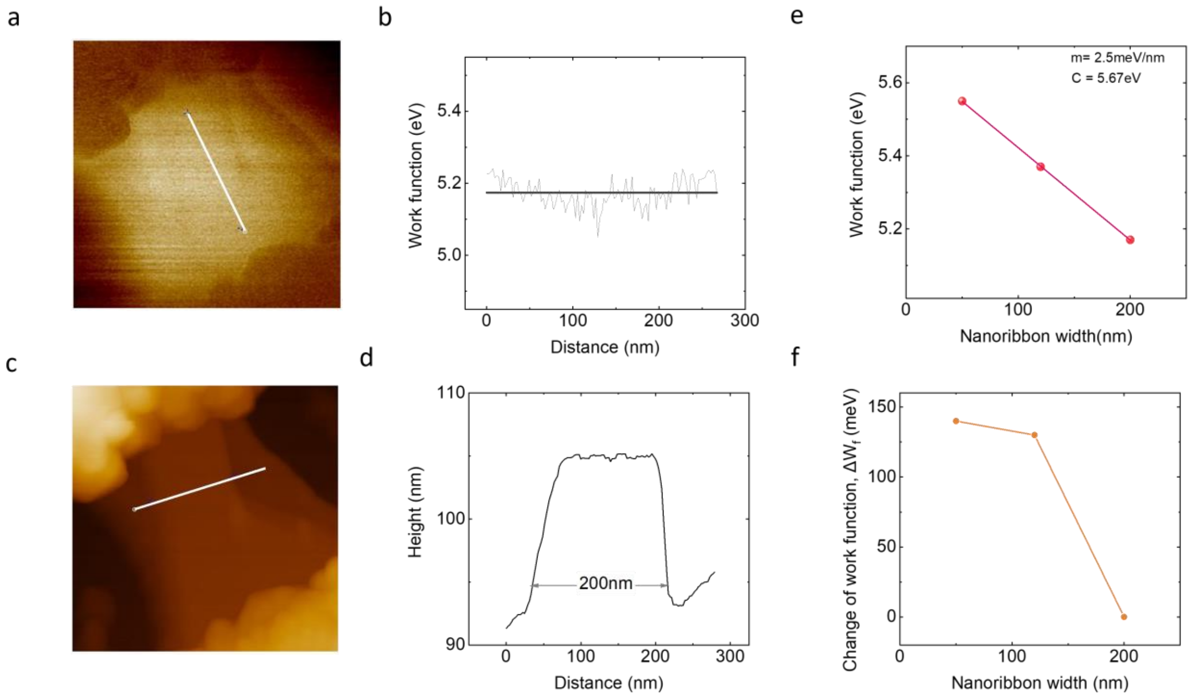

Figure S8: Width-dependent Kelvin probe force microscopy (KPFM) analysis. (a) Kelvin probe force microscopy (KPFM) image of an etched WS_2 nanoribbon with wider nanoribbon section. **(b)** The estimated work function of the etched wider WS_2 nanoribbon with narrower and wider sections across the white line in Figure S8a. For this nanoribbon channel, the work function (W_f) for the narrow channel is about 5.17 eV, which is bit lower from the estimated W_f of the nanoribbon presented in Figure 2h and Figure S7 because this nanoribbon is much wider than the one presented in other figures. **(c)** AFM image of the nanoribbon that is used for KPFM analysis. **(d)** The height profile (along the white line in (c) shows the thickness of the WS_2 flake is about 15 nm and the nanoribbon is about 200nm wide. **(e)** Estimated work function as a function of nanoribbon width along with linear fitting (solid line). The narrowest nanoribbon shows higher W_f and widest ribbon shows lowest W_f values. To be noted, lower W_f indicates higher n-type doping in the channel, as expected for the wider ribbon and higher W_f indicates depletion of carriers in the channel. **(f)** Change of work function (ΔW_f) between the wider and narrower regions in the nanoribbons. The ΔW_f decreases with increasing the channel width. This analysis confirms our findings of the electrical measurements in this article that the narrower channel shows depletion of carriers in comparison to the wider regions, and a depletion region emerges between the narrow and wider nanoribbon regions.

Schottky barrier height, mobility and channel current in nanoribbon FETs

In the thermionic-emission model[2–4], channel current I_{ds} can simply be expressed as $I_{ds}=A^{*}AT^{2}\exp(-e\Phi_b/k_bT)\,[\exp(eV_{ds}/k_bT)-1]$, where A*, A, e, V_{ds}, and k_b are the Richardson constant, the area of the contact, the elemental charge, the source-drain voltage, and the Boltzmann constant, respectively. Temperature-dependent IV properties of nanoribbons with 70 nm and 47 nm channel widths are depicted in Fig. S3b and Fig. S3d, respectively. The reciprocal temperature (1000/T) dependence of the $\ln(I_{ds}/T^2)$, also known as Arrhenius plots are delineated in Supplementary Fig. S3c and Fig. S3e for nanoribbons with 70 nm and 47 nm channels, respectively, at various V_{ds}. The

corresponding slope, S, for each V_{ds} was extracted from the Arrhenius plots. The intercept of the slope, S_0, yielded the barrier height, Φ_b (Fig. 2i). From the expression $S_0 = -e\Phi_b/1000k_b$, the barrier height is estimated.

In the following, we have included the Schottky barrier (SB) height estimation at different V_g with V_{ds} = 5 V in the nanoribbon FETs with channel widths of 47 nm, 34 nm, 28 nm and 18 nm. The nanoribbon FETs with channel widths 47 nm and 34 nm show a similar trend of the SB height. In these nanoribbon FETs, the SB heights are higher (200 meV) at V_g <0 V and decreases sharply with increasing V_g. At V_g beyond 25 V, the adapted model for SB height calculations deviates from the thermionic emission transport, which limits the estimation of the SB height at V_g>25 V. The estimated SB height at various V_g for the 28 nm wide nanoribbon FET shows a trend similar to the 47 nm and 34 nm wide nanoribbon FETs but at different V_g range because of the narrow channel effect. The calculation of SB barrier height for the 18 nm wide nanoribbon FET is performed at V_g>70 V due to the narrow channel effect and deviation to follow the thermionic emission model. The FETs show SB barrier heights of about 150 meV with V_{ds} = 5 V, consistent with the reported value for the 47 nm wide nanoribbon in Figure 2i in the main manuscript. The barrier heights that are shown in Figure 2i in the main manuscript are estimated at V_g = 80 V with lower V_{ds} values, compatible with SB calculations using the thermionic emission model[2–4].

We estimated the Schottky barrier height of about 74 meV and 167 meV for 70 nm and 47 nm nanoribbon channels, respectively. The difference in the contact barrier height between 47 nm and 70 nm wide channels is about 90 meV with Ti/Au electrodes (bias voltage), deduced from the thermionic emission model. On the other hand, the KPFM measurements directly show the difference in work functions between the narrower and wider junctions in etched WS_2. We found the difference in the work function (Fermi level position) is about ΔW_f = 130 meV across the wider and narrower part of the etched WS_2. We further observe a change in work function, ΔW_f = 140 meV in a 50 nm nanoribbon junction, and no change in the W_f in the widest (200 nm) nanoribbon junction.

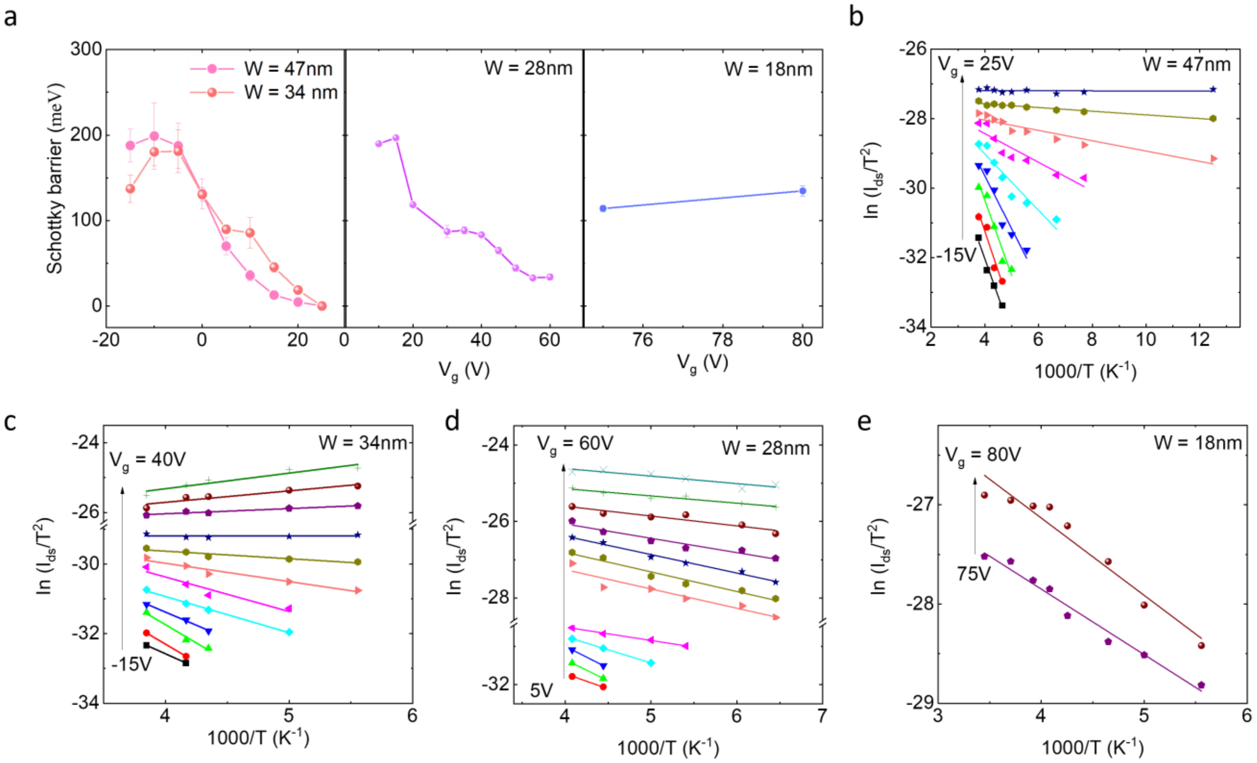

Figure S9: Width-dependent Schottky barrier height in nanoribbon FETs. (a) Estimated Schottky barrier (SB) height at different V_g with V_{ds} = 5 V in the nanoribbon FETs with channel widths of 47 nm, 34 nm, 28 nm and 18 nm. The nanoribbon FETs with channel widths 47 nm and 34 nm show a similar trend of the SB height (left panel). In these nanoribbon FETs, the SB height is higher (200 meV) at V_g <0V and decreases sharply with increasing V_g. At V_g beyond 25 V, the adapted model for SB height calculations deviates from the thermionic emission transport, which limits the estimation of the SB height at V_g>25 V. The estimated SB height at various V_g for the 28nm wide nanoribbon FET (middle panel) shows a trend similar to the 47 nm and 34 nm wide nanoribbon FETs but at different V_g ranges because of the narrow channel effect. The calculation of SB barrier height for the 18nm wide nanoribbon FET (right panel) is performed at V_g>70 V due to the narrow channel effect and deviation to follow the thermionic emission model. **(b), (c), (d)** and **(e)** Arrhenius plots of ln (I_{ds}/T^2) with 1000/T at different V_g at V_{ds} = 5 V in the nanoribbon FET with channel width 70 nm, 34 nm, 27 nm and 18 nm. In summary, the FETs show SB barrier heights of about 150 meV with V_{ds} = 5 V, consistent with the reported value for the 47 nm wide nanoribbon in Figure 2i in the main manuscript. The barrier heights that are shown in Figure 2i in the main manuscript are estimated at V_g = 80 V with lower V_{ds} values, compatible with SB calculations using the thermionic emission model[2–4].

Fabrication of WS_2 nanoribbon FET with 9 nm and 5 nm channel width

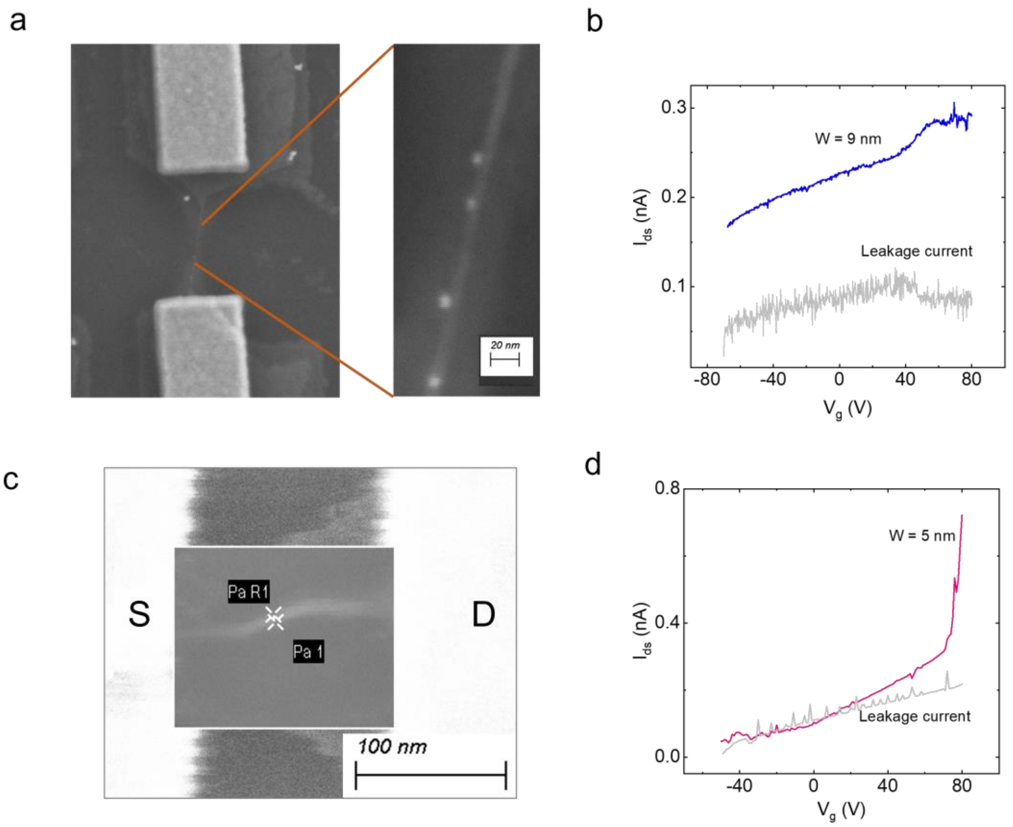

Figure S10: WS_2 nanoribbon FETs with 9 nm and 5 nm channel width. (a) SEM image of the fabricated WS_2 nanoribbon FET with 9 nm channel width. **(b)** Transfer properties of the FET with 9 nm channel width at V_{ds}=5 V. The gray data shows the leakage current between the contact and the gate electrode. **(c)** SEM image of a fabricated WS_2 nanoribbon FET with about 5 nm channel width in another fabrication batch, which is different from the devices presented in the main manuscript. The SEM image is a bit distorted due to scanning issues in the tool, but the WS_2 channel is conspicuous. **(d)** The transport properties (I_{ds} vs. V_g) of the FET with 5 nm channel width by applying V_{ds}=5 V. The channel length is L=200 nm and threshold voltage $V_{th} \approx 73$ V. It is to be mentioned that FET with 5 nm channel (Fig. S10d) exploded when we applied higher gate voltage due to oxide breakdown of the gate dielectric, SiO_2.

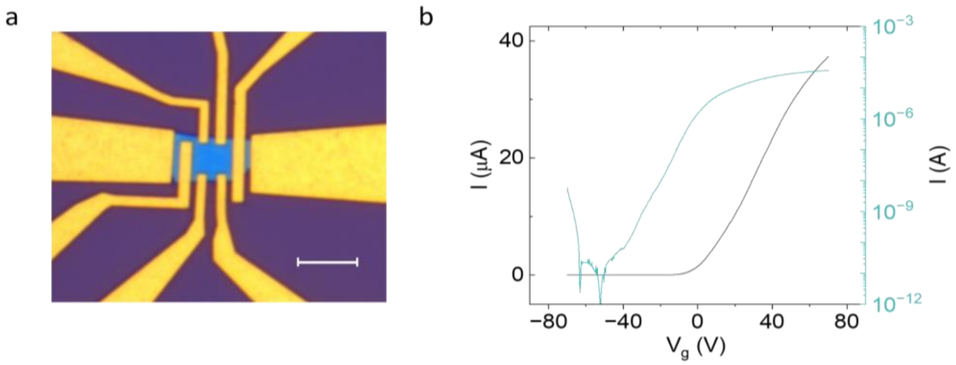

Figure S11: Transport properties of a pristine WS_2 FET. (a) Optical microscopy image of a fabricated multi-channel WS_2 nanoribbon FET with a scale bar of 5 μm. (b) Transport properties (I_{ds} vs. V_g) at V_{ds} = 1 V in a pristine WS_2 FET. The channel width, W = 4.4 μm, and length, L = 3.43 μm.

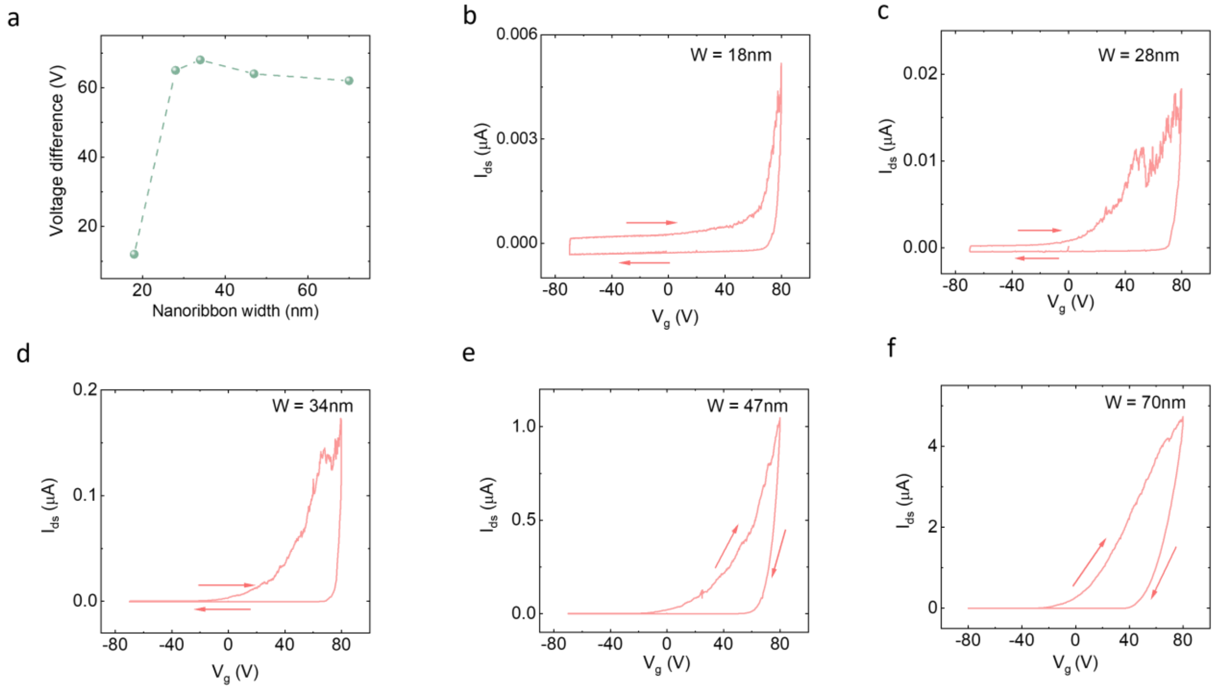

Figure S12: Width-dependent gate voltage hysteresis between the forward and backward sweeping directions in nanoribbon WS_2 FET. (a) Voltage difference of the applied gate-voltages for the forward and backward sweeping directions of the nanoribbon FETs in various widths. The voltage difference is estimated at the I_{ds} = 1 nA at room temperature. The voltage difference is lower for the FET with 18 nm channel width and almost indifferent for the other nanoribbon FETs. **(b, c, d, e, f)** I_{ds} vs. V_g for V_{ds} = 5 V for forward and backward V_g sweeping directions (denoted by the arrows) in the nanoribbon FETs with channel widths 18 nm, 28 nm, 34 nm, 47 nm, 70 nm, respectively.

Transport properties of WS_2 nanoribbon FETs at different temperatures

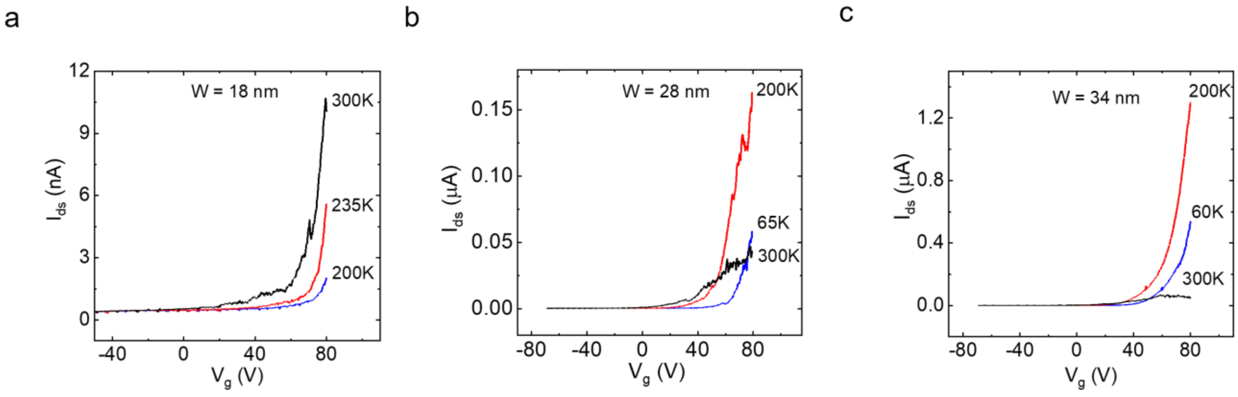

Figure S13: Transport properties of WS_2 nanoribbons at different temperatures. **(a,b,c)** Measured transfer properties of the WS_2 FETs with 18 nm, 28 nm and 34 nm channel width at different temperatures with V_{ds}=5 V.

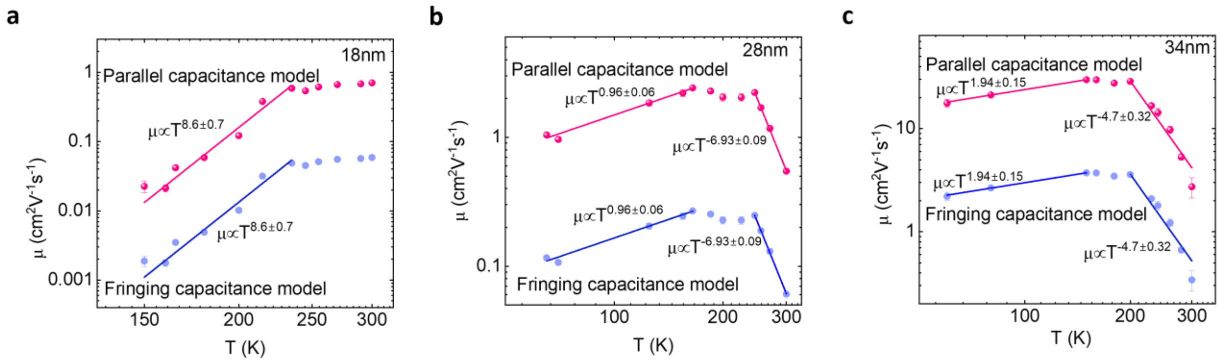

Figure S14: Mobility estimation from parallel and fringing capacitance models. (a, b, c) Estimated mobility μ with parallel (pink dots) and fringing (blue dots) capacitance models at different temperatures T of the WS_2 nanoribbon FETs with 18 nm, 28 nm and 34 nm channel widths along with the power-law fitting with $\mu \propto T^{\gamma}$(solid line) for different temperature ranges. If we used the fringing capacitance model to calculate mobility, the value of the mobility decreases about 8-12 times depending on the width of the nanoribbon. However, the trend of the estimated mobility with temperature and hence, the deduction of scattering mechanisms remain the same, as it is observed in the figures.

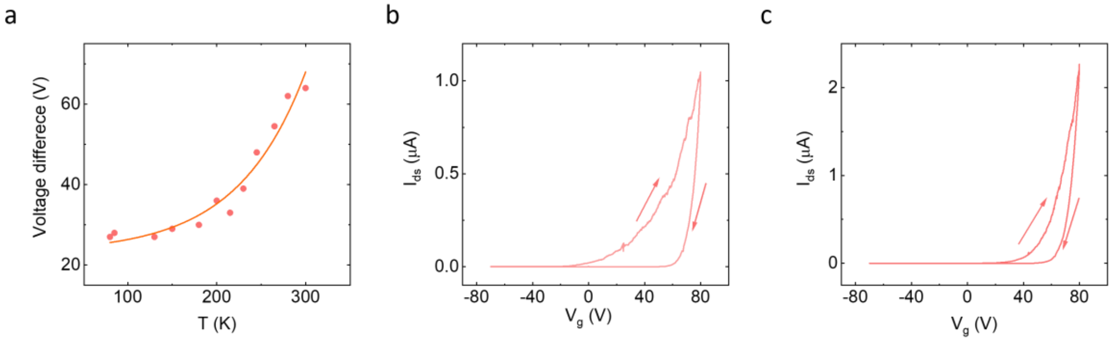

Figure S15: Temperature-dependent gate voltage hysteresis between the forward and backward sweeping directions in nanoribbon WS_2 FET. (a) Voltage difference of the applied gate-voltages (V_g) for the forward and backward sweeping directions of the nanoribbon FET at various temperatures. The voltage difference is estimated at V_{ds} = 5 V and the solid line shows the exponential fits as guides to the eye. **(b, c)** I_{ds} vs. V_g for forward and backward V_g sweeping directions (denoted by the arrows) in the nanoribbon FET with channel width 47 nm at 300 K and 80 K, respectively.

Work function calculation process from KPFM measurement

In the KPFM experiment, one measures the Contact Potential Difference (CPD) between the work functions of the tip (W_{tip}) and the surface/sample (W_{sample}):

$$V_{CPD} = (W_{tip} - W_{sample})/e \quad \text{(S1)}$$

Where, e is the charge of electron. Therefore, in order to obtain the work function of the sample, we calibrated the work function of the tip. This is done by measuring on a known sample, such as oriented pyrolytic graphite (HOPG) ($W_{HOPG} = 4.48\ eV$):

$$W_{tip} = eV_{CPD2} + W_{HOPG} = eV_{CPD2} + 4.48\ V \quad \text{(S2)}$$

By combining equations (S1) and (S2), one can calculate the work function of the unknown sample. We found V_{CPD2} = 1V for our Platinum-Iridium coated tip with HOPG.

Computational Details of Density Functional Theory Calculation

Our calculations are performed using the spin-polarized density functional theory (DFT) as implemented in the Vienna ab initio simulation package (VASP)[5,6]. The electron-ion interaction is described by the projector augmented wave (PAW) method[7]. For the exchange-correlation functional we employ the generalized gradient approximation (GGA), suggested by Perdew, Burke, and Ernzerhof (PBE)[8], and van der Waals corrections are included using the Grimme-D3 method[9]. We model WS_2 nanoribbons with zigzag edges, featuring a 3x periodicity and comprising 10 zigzag chains. To prevent fictitious interactions between neighboring cells, we embed vacuum layers larger than 15 Å perpendicular to the edges. For the atomic relaxations and density of states calculations, the Brillouin

zone is sampled using (6x1x1) and (10x1x1) k-point grids, respectively, and a kinetic energy cutoff of 500 eV is implemented.

Results of Density Functional Theory Calculation

As shown in Fig. S16a and Fig. S16b, WS_2 zigzag nanoribbons with non-saturated edges exhibit a ferromagnetic metallic behavior which agrees with previously reported theoretical calculations[10,11]. For semiconducting materials like WS_2 nanosheets, dangling bonds at the edges need to be eliminated to preserve the band gap. Their elimination can be achieved either by extrinsic or intrinsic atoms via proper reconstruction. For the case of WS_2, each W donates 4 electrons and each S gains 2 electrons. For bulk 1H-WS_2, W is bonded to six S atoms and therefore W donates 4/6=2/3 electrons to a neighboring S atom, whereas S is bonded to three W atoms and therefore S gains 2/3 electrons from a neighboring W atom. For a formula unit of 1H-WS_2, the net charge is found to be (6×2/3)-2×(3×2/3)=0. The zero net charge is not observed in the zigzag edges and the charge balance needs to be achieved through proper reconstruction. Following the work by Lucking et al.[12], we consider nanoribbons with various S and W edge reconstructions that fulfill the Electron Counting Model and we take into account that W is a transition metal that can also exist in valence states of 5+ or 6+. Regarding the S edge, three different reconstructions are considered, namely 1S_2 (Fig. S16d), where one pair of S atoms at the edge come close to each other to form one dimer, 2S_2 (Fig. S16g), where two pairs of S atoms at the edge come close to each other to form two dimers, 1V_s (Fig. S16j) where one S atom at the edge is removed forming a S vacancy. Regarding the W edge, we consider a two-electron deficiency, meaning that either two W atoms alter their valence to 5+ or one W atom alters its valence to 6+. In particular, we adopt the so-called 3S reconstruction, where three S atoms are placed at the W-terminated edge.

The DOS (Fig. S16e, Fig. S16h, Fig. S16k) and corresponding partial DOS (Fig. S16f, Fig. S16i, Fig. S16l) of the nanoribbons with various reconstructions are computed and compared with the DOS of the infinite single-layer system (Fig. S16c). For the sake of comparison, a proper alignment of the states has been done, and the Fermi level is computed by considering (ECBM+EVBM)/2. As observed in Fig.S16, all considered configurations exhibit sizable band gaps ranging from about 0.4 to 0.6 eV within the PBE level. The partial DOS reveals that the states near the valence and conduction band extrema originate from the atoms close to the edges. For all cases, the middle of the band gap, so the estimated Fermi level position, is below the middle of the band gap of the monolayer sheet. This agrees well with the p-type character of the nanoribbons observed in experiments.

Overall, it is evident from the DFT that it shows a metallic band structure in the nanoribbon; however, the reconstructed nanoribbon shows a finite bandgap opening. Furthermore, the reconstructed nanoribbons exhibit a shift of the Fermi level position towards the valence bands, compared to the pristine infinite WS_2, resulting in electron depletion. These findings are compatible with the experimentally observed electronic properties of the nanoribbons, where nanoribbon depicts electron depletion compared to the wider WS_2 channel.

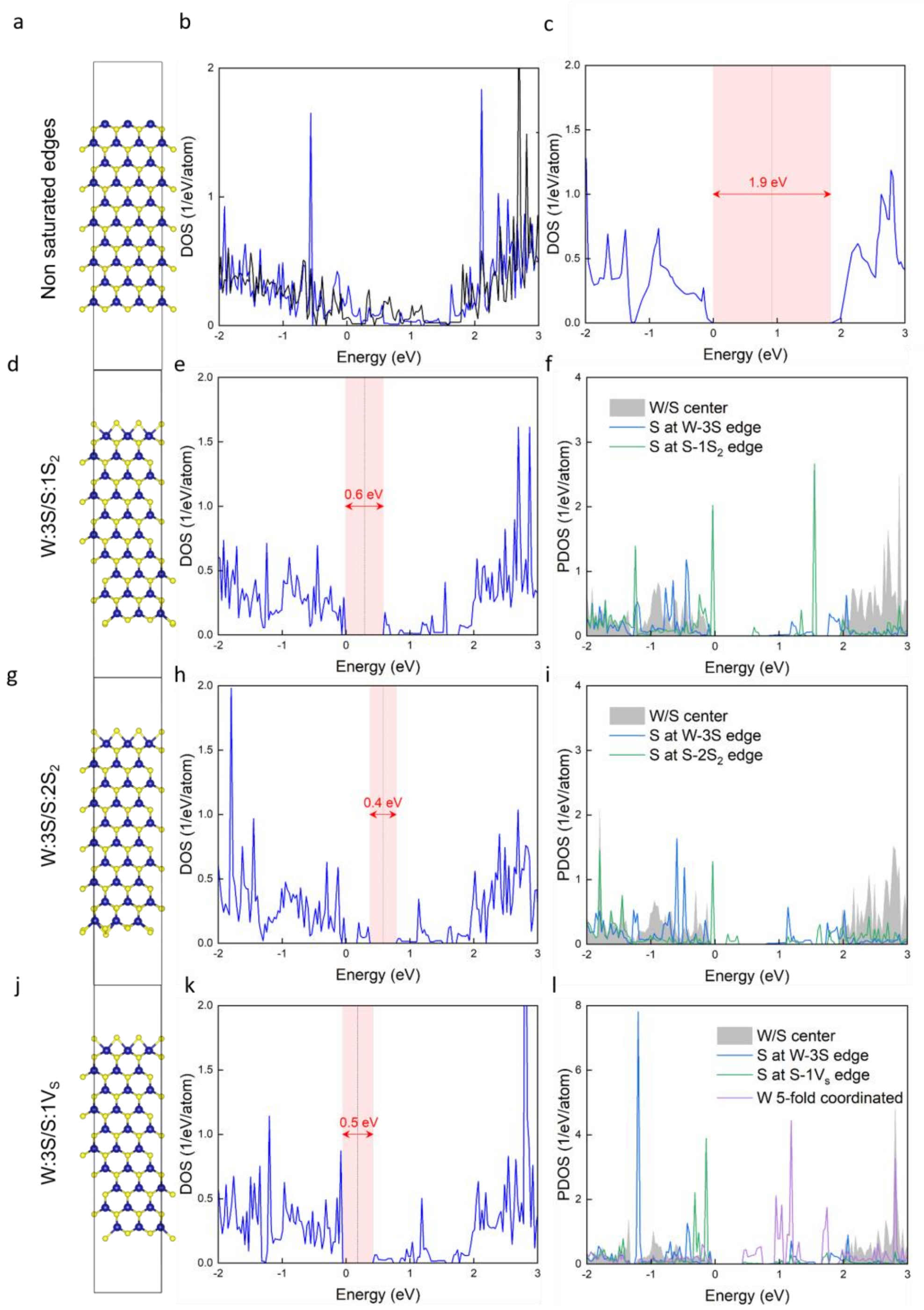

Figure S16: Calculated comparative analysis of WS_2 nanoribbons with various edge reconstructions. The left column **(a, d, g, j)** shows the atomic structures of the nanoribbons. The middle column **(b, e, h, k)** shows the electronic density of states (DOS) of the ribbons, with shaded areas indicating the band gap. The right column contrasts the total DOS of the pristine WS_2 nanosheet (c), with the partial density of states (PDOS) of the nanoribbons with various edge reconstructions (f, i, l). The midpoint between the occupied and unoccupied states is indicated by dashed lines and all DOS are aligned appropriately for comparative analysis of the states. Blue and yellow spheres correspond

to W and S atoms, respectively. For the DOS of the nanoribbon with non-saturated edges, blue and black lines refer to spin-up-like and spin-down-like states, respectively.

For the nanoribbon with the largest band gap (W:3S/S:1S_2), we examined the oxidation of the edges. We studied various models where O molecules were placed at different positions and different orientations (vertical or horizontal). As shown in Fig.S17, we found that oxygen molecules (red atoms in Fig. S17a) dissociate and bind to S and/or W atoms. For the energetically favorable oxidized structure, the DOS and PDOS were computed and compared with the DOS of the single-layer system in the absence of oxygen molecules. Similar to our previous analysis (shown in Figure S16), the middle of the band gap (estimated Fermi level position) is below the middle of the band gap of the monolayer sheet. Hence, we found that the reconstruction and oxidation of WS_2 nanoribbon edges lower the Fermi level position towards the valance bands compared to the pristine WS_2 wider nanosheet, which is realized in light of observed carrier depletion and shift of V_{th} in the transport measurements.

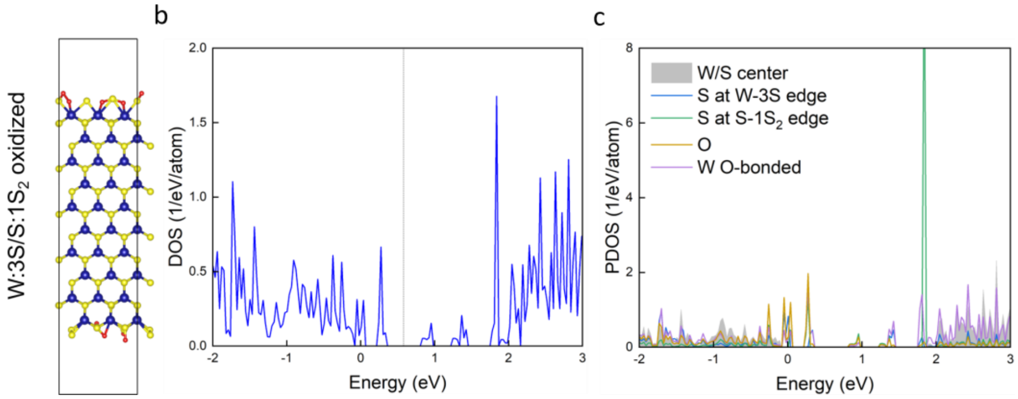

Figure S17: Calculated oxidation effect of the nanoribbon edges. **(a)** The atomic structure of the WS_2 nanoribbon with oxygen atoms bonded at the edges. **(b)** and **(c)** The electronic density of states (DOS) of the oxidized nanoribbon and the right panel shows the corresponding partial density of states (PDOS), respectively. The midpoint between occupied and unoccupied states is indicated by dashed lines and all DOS plots are aligned appropriately for comparative analysis of the states. Blue, yellow, and red spheres correspond to W, S, and O atoms, respectively.

Supplementary References

1. Munkhbat, B. *et al.* Transition metal dichalcogenide metamaterials with atomic precision. *Nat. Commun.* **11**, 4604 (2020).
2. Chen, J.-R. *et al.* Control of Schottky Barriers in Single Layer MoS 2 Transistors with Ferromagnetic Contacts. *Nano Lett.* **13**, 3106–3110 (2013).
3. Dankert, A., Langouche, L., Kamalakar, M. V. & Dash, S. P. High-performance molybdenum disulfide field-effect transistors with spin tunnel contacts. *ACS Nano* **8**, 476–482 (2014).
4. Dankert, A. & Dash, S. P. Electrical gate control of spin current in van der Waals heterostructures at room temperature. *Nat. Commun.* **8**, 1–6 (2017).
5. Kresse, G. & Furthmüller, J. Efficiency of ab-initio total energy calculations for metals and semiconductors using a plane-wave basis set. *Comput. Mater. Sci.* **6**, 15–50 (1996).
6. Kresse, G. & Furthmüller, J. Efficient iterative schemes for ab initio total-energy calculations using a plane-wave basis set. *Phys. Rev. B - Condens. Matter Mater. Phys.* **54**, 11169–11186 (1996).

7. Blöchl, P. E. Projector augmented-wave method. *Phys. Rev. B* **50**, 17953–17979 (1994).
8. Perdew, J. P., Burke, K. & Ernzerhof, M. Generalized gradient approximation made simple. *Phys. Rev. Lett.* **77**, 3865–3868 (1996).
9. Grimme, S., Antony, J., Ehrlich, S. & Krieg, H. A consistent and accurate ab initio parametrization of density functional dispersion correction (DFT-D) for the 94 elements H-Pu. *J. Chem. Phys.* **132**, 154104 (2010).
10. López-Urías, F. *et al.* Electronic, magnetic, optical, and edge-reactivity properties of semiconducting and metallic WS2 nanoribbons. *2D Mater.* **2**, 015002 (2015).
11. Ao, L. *et al*. Tunable electronic and magnetic properties of arsenene nanoribbons. *RSC Adv.* **7**, 51935–51943 (2017).
12. Lucking, M. C., Bang, J., Terrones, H., Sun, Y. Y. & Zhang, S. Multivalency-induced band gap opening at MoS2 edges. *Chem. Mater.* **27**, 3326–3331 (2015).
